# 16S rRNA Gene Amplicon Sequencing of Gut Microbiota Affected by Four Probiotic Strains in Mice

**DOI:** 10.3390/vetsci10040288

**Published:** 2023-04-12

**Authors:** Jianwei Ren, Fang He, Detao Yu, Hang Xu, Nianfeng Li, Zhi Cao, Jianxin Wen

**Affiliations:** College of Veterinary Medicine, Qingdao Agricultural University, Qingdao 266109, China; a17852022250@outlook.com (J.R.); hefang1030@126.com (F.H.);

**Keywords:** probiotics, *L. acidophilus*, *L. plantarum*, *B. subtilis*, *E. faecalis*, 16S rRNA gene amplicon sequencing

## Abstract

**Simple Summary:**

The overuse of antibiotics has led to an increase in resistant bacteria and unnecessary culture contamination. As one of the best green pollution-free antibiotics, probiotics and their preparations have become research hotspots. The different effects of different probiotics on gut microbiota are still unclear. In this study, the gut microbiota of mice treated with *Lactobacillus acidophilus*, *Lactobacillus plantarum*, *Bacillus subtilis* and *Enterococcus faecalis* for 14 days was assessed used 16S amplificon sequencing. The results showed that the four probiotics caused changes in the composition and structure of the gut microbiota in mice, but they did not cause changes in the diversity of the gut microbiota. These results provide a strong basis for the preparation of probiotics and theory regarding their targets.

**Abstract:**

Probiotics, also referred to as “living microorganisms,” are mostly present in the genitals and the guts of animals. They can increase an animal’s immunity, aid in digestion and absorption, control gut microbiota, protect against sickness, and even fight cancer. However, the differences in the effects of different types of probiotics on host gut microbiota composition are still unclear. In this study, 21-day-old specific pathogen-free (SPF) mice were gavaged with *Lactobacillus acidophilus* (La), *Lactiplantibacillus plantarum* (Lp), *Bacillus subtilis* (Bs), *Enterococcus faecalis* (Ef), LB broth medium, and MRS broth medium. We sequenced 16S rRNA from fecal samples from each group 14 d after gavaging. According to the results, there were significant differences among the six groups of samples in Firmicutes, Bacteroidetes, Proteobacteria, Bacteroidetes, Actinobacteria, and Desferribacter (*p* < 0.01) at the phylum level. *Lactobacillus*, *Erysipelaceae Clostridium*, *Bacteroides*, *Brautella*, *Trichospiraceae Clostridium*, *Verummicroaceae Ruminococcus*, *Ruminococcus*, *Prevotella*, *Shigella*, and *Clostridium Clostridium* differed significantly at the genus level (*p* < 0.01). Four kinds of probiotic changes in the composition and structure of the gut microbiota in mice were observed, but they did not cause changes in the diversity of the gut microbiota. In conclusion, the use of different probiotics resulted in different changes in the gut microbiota of the mice, including genera that some probiotics decreased and genera that some pathogens increased. According to the results of this study, different probiotic strains have different effects on the gut microbiota of mice, which may provide new ideas for the mechanism of action and application of microecological agents.

## 1. Introduction

The gut microbiota and the host complement each other and are indispensable in the intestinal tract of animals [1]. The gut microbiota is a microbial community formed when animals are born, and it is transmitted from the mother and subsequently influenced by the external environment [2]. In the gut of an animal, the gut microbiota is maintained in a balanced state. In such a balanced state, the microbial community interacts with each other and the host, so that the animal can maintain a healthy body condition [3]. The symbiotic interactions between resident microorganisms and the gastrointestinal tract significantly contribute to maintaining gut homeostasis. Disorder of the gut microbiota can cause a variety of host-related diseases [4], such as functional gastrointestinal diseases [5], intestinal infectious diseases [6], liver diseases [7], obesity and metabolic syndrome [8], diabetes mellitus [9], autism [10], etc.

There are many factors that can influence gut microbiota. Diet plays a key role in the regulation of gut microbiota composition. Different diets can cause different changes in the composition of gut microbiota [4]. Antibiotic use leads to long-term consequences such as reduced microbial diversity, disproportion, and increased expansion of the opportunistic pathogens *Escherichia* and *Klebsiella* [11]. With the entry of probiotics and prebiotics into the public eye, their potential role in reshaping the gut microbiota to enhance gut health has been gradually established [12]. Because of their green and pollution-free characteristics, they can be used as effective substitutes for antibiotics and have been widely used in the animal rearing industry. Probiotics can produce active substances that have positive effects on the host, promote digestion and nutrient absorption, regulate the gut microbiota, improve animal immunity, prevent and treat diseases, and even resist cancer [13]. Some probiotics can effectively inhibit the growth of pathogenic bacteria, promote gut peristalsis, stimulate the immune system, and strengthen the gut mucosal barrier in the gastrointestinal tract of animals [14].

As commonly used probiotics, *Lactobacillus* and its subspecies are widely used in food processing, preservation, and fermentation. Because most *Lactobacillus* have the biological characteristics of acid and bile salt tolerance, they can utilize their good characteristics in the intestine [15,16,17]. *L. acidophilus* and *L. plantarum* are currently widely used in food as safe probiotics [18,19]. *B. subtilis* [20] can produce spores, enabling it to tolerate gastric fluid and bile salts and have a probiotic effect in the gut. *E. faecalis* can tolerate relatively severe environments, such as pH 9.6 and high concentrations of salt, and is often used in fermented products [21]. Therefore, good stability will be maintained during the processing, storage, and transportation of probiotic preparations [22,23,24].

As for *L. acidophilus*, *L. plantarum*, *B. subtilis*, and *E. Faecalis*, which act as probiotics on the gut in the body, the differences in the effects on gut microbiota are still unclear. In this study, mice were orally gavaged with a fresh bacterial culture of four probiotics with specific concentrations, and changes in the gut microbiota of the mice were analyzed.

## 2. Materials and Methods

Table 1 shows the sources and storage locations of *L. plantarum*, *L. acidophilus*, *B. subtilis*, and *E. faecalis*; the Kunming mice (bought from Qingdao Daren Fucheng Co., Ltd., Qingdao, China) in this study were fifteen days old and pre-fed for one week (21 d old) for the test. The animal experiments performed in this study strictly followed the national guidelines for experimental animal welfare announced by the Ministry of Science and Technology of the People’s Republic of China in 2006 (Guiding Opinions on Kindly Treating Laboratory Animals. Relevant link: https://www.most.gov.cn/xxgk/xinxifenlei/fdzdgknr/fgzc/gfxwj/gfxwj2010before/201712/t20171222_137025.html (accessed on 3 April 2021)) and were approved by the Animal Welfare and Research Ethics Committee at Qingdao Agricultural University, Shandong, China (Approval NO: 2021-56). An LB broth medium and an MRS broth medium (Qingdao Haibo Biotechnology Co., Qingdao, China) were used in the study.

*L. plantarum*, *L. acidophilus*, *B. subtilis*, and *E. faecalis* were incubated for 6 h, 8 h, 10 h, 12 h, and 14 h. *L. plantarum* and *L. acidophilus* were incubated in a warm oven with an MRS broth, while *B. subtilis* and *E. faecalis* were incubated in a shaker with an LB broth at 180 rpm/min. The turbidity of each bacterium was measured at different incubation times (the turbidity was measured by a WGZ-XT intelligent bacterial turbidity meter, Hangzhou Qiwei Instrument Co., Hangzhou, China).

Thirty-six mice of approximately the same size and weight, eighteen males and eighteen females, were selected and divided equally into six groups.

Gavage was administered to each group of mice separately and continued for 14 d. *L. plantarum* (Lp) and *L. acidophilus* (La) were selected as the test groups where the MRS broth medium was selected as the control group (MRS). *B. subtilis* (Bs) and *E. faecalis* (Ef) were selected as the test groups where the LB broth medium (LB) was selected as the control group. Bacterial liquid and broths were administered in quantities of 0.1 mL/animal and gavage took place at 17:00 BST daily.

The experiment was conducted for 14 d, and at the end of the experiment, fresh feces from each group of mice were collected separately and transferred to a −80 °C freezer for storage.

Total microbial genomic DNA samples were extracted using an OMEGA Soil DNA Kit (D5625-01) (Omega Bio-Tek, Inc.; Norcross, GA, USA) following the manufacturer’s instructions, and they were stored at −20 °C prior to further assessment. The extracted DNA was determined in terms of quantity and quality using a NanoDrop ND-1000 spectrophotometer (Thermo Fisher Scientific, Waltham, MA, USA) and agarose gel electrophoresis, respectively.

The quality DNA was sent to Personal Biotechnology Co., Ltd. (Shanghai, China) for sequencing of the bacterial 16S V3V4 region.

Sequence quality control and splicing were performed using the DADA2 method. QIIME2(2019.4) and R software were used to analyze the taxonomic composition, α-diversity, and β-diversity of the samples.

## 3. Results

### 3.1. Bacterial Concentration Results

The results in Table 2 show that the colony concentration of *L. plantarum* and *L. acidophilus* still grew rapidly after 6 h of incubation. The proliferation of bacteria started to slow down after 8 h of incubation. The growth of *B. subtilis* and *E. faecalis* slowed down after 6 h of incubation. The proliferation of bacteria almost stopped after 8 h of incubation. In this study, the selected bacterial liquid was cultured for 8 h, and 0.1 mL was used, with the concentration of bacterial liquid being ≥10^8^ CFU/mL.

### 3.2. Sequence Processing

Table 3 shows the basic sequencing information of the six groups of samples in this study. The number of original sequences and the number of sequences after quality control and trimming were included. After quality control and denoising, the effective sequences obtained were as follows: La group 69,469; Lp group 119,571; MRS group 51,734; Bs group 52,800; Ef group 51,550; LB group 72,382. The number of sequences was as follows: La 63,712; Lp 109,512; MRS 47,739; Bs 50,650; Ef 47,408; LB 69,136. The amount of data after removing low-quality sequences were as follows: La group 44,755; Lp group 72,120; MRS group 34,229; Bs group 36,812; Ef group 36,800; LB group 49,334.

**Figure 1:**
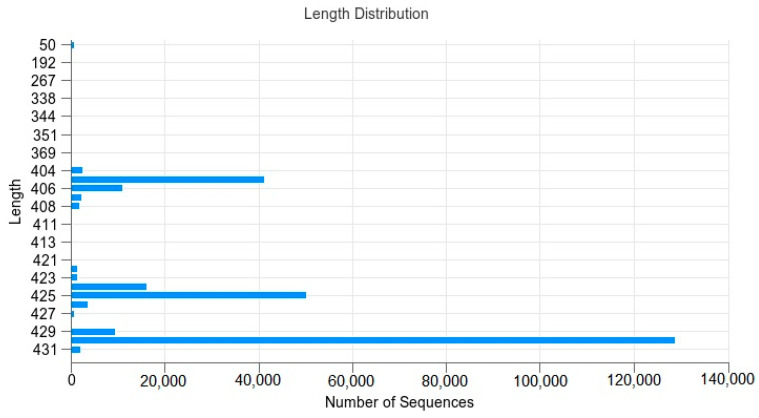
Sequence–length distribution: The abscissa is the number of sequences, and the ordinate is the length of the sequence.

Figure 1 shows the distribution of sequences in the sample as well as their lengths.

The results show that the sequence lengths were mainly distributed as follows: 405 bp; 406 bp; 424 bp; 425 bp; 429 bp; 430 bp. The numbers were 41,115; 11,065; 16,081; 50,169; 9203; 128,783.

### 3.3. Four Probiotics Affect Gut Microbiota Diversity in Mice

**Figure 2:**
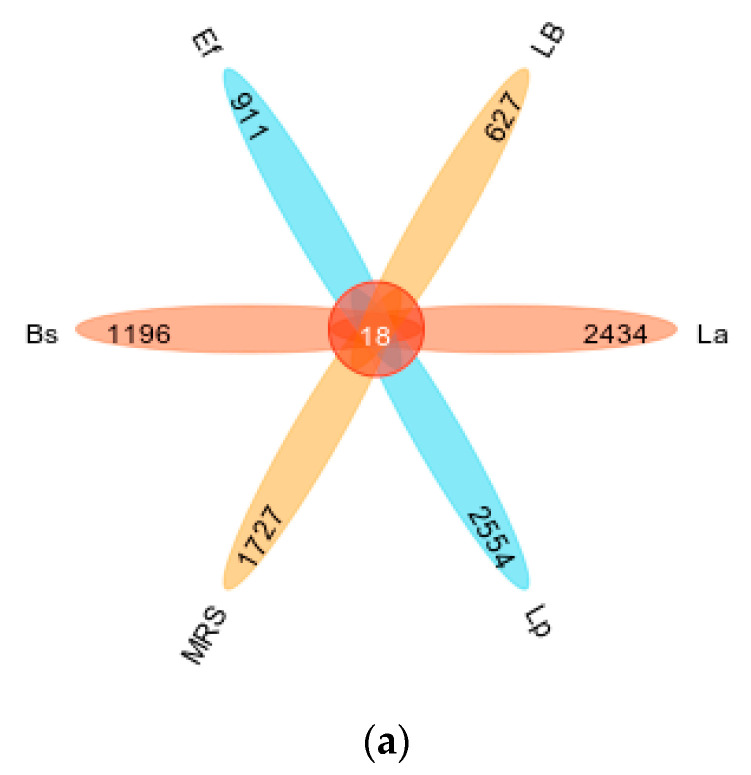

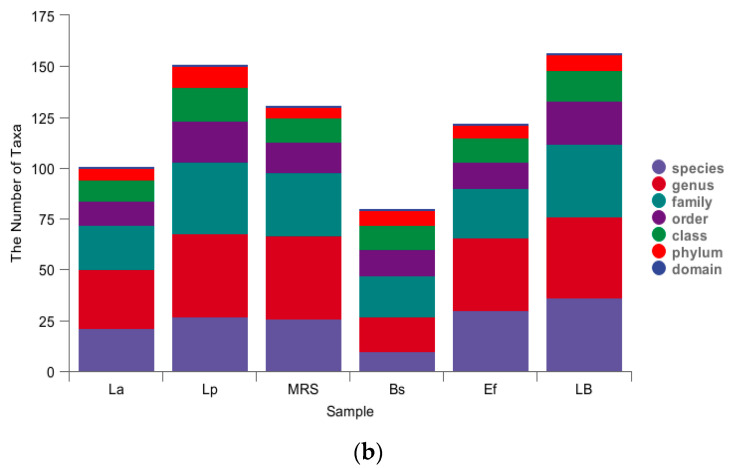
Classification unit number statistics: (**a**) petal map of taxonomic unit statistics (indicating the number of OTUs unique and common to each of the six groups Lp, La, MRS, Bs, Ef, and LB); (**b**) histogram of taxonomic unit statistics (indicating the number of taxonomic units contained in each of the six groups Lp, La, MRS, Bs, Ef, and LB at the seven taxonomic levels of domain, phylum, order, family, genus, and species, respectively, with unclassified, uncultured, uncultivated, unknown, metagenome, etc., units removed).

As shown in Figure 2a, the mean number of OTUs annotated to the Lp and La groups was higher than the other groups; LB, as a control group for the Bs and Ef groups, contained the lowest number of OTUs (Figure 2a: petal plot Lp 2572; La 2452; MRS 1745; Bs 1214; Ef 929; LB 645). The specific composition of the microbial community in each sample at each taxonomic level could be obtained by counting the ASV/OTU after flat sampling, as shown in Figure 2b. In each of the six groups, different numbers of taxonomic units were detected. The La, Lp, and MRS groups contained 101, 151, and 131 taxonomic units, respectively; the Bs, Ef, and LB groups contained 80, 122, and 157 OTUs, respectively. Among them, the La group contained 6 phyla, 10 classes, 12 orders, 22 families, 29 genera, and 21 species. The Lp group contained 10 phyla, 17 classes, 20 orders, 35 families, 41 genera, and 27 species. There were 5 phyla, 12 classes, 15 orders, 31 families, 41 genera, and 26 species in the MRS Group. There were 7 phyla, 12 classes, 13 orders, 20 families, 17 genera, and 10 species in the Bs group. The Ef group contained 7 phyla, 12 classes, 13 orders, 24 families, 36 genera, and 30 species. The LB group contained 8 phyla, 15 classes, 21 orders, 36 families, 40 genera, and 36 species.

### 3.4. Four Probiotics Alter the Distribution of Gut Microbiota in Mice

**Figure 3:**
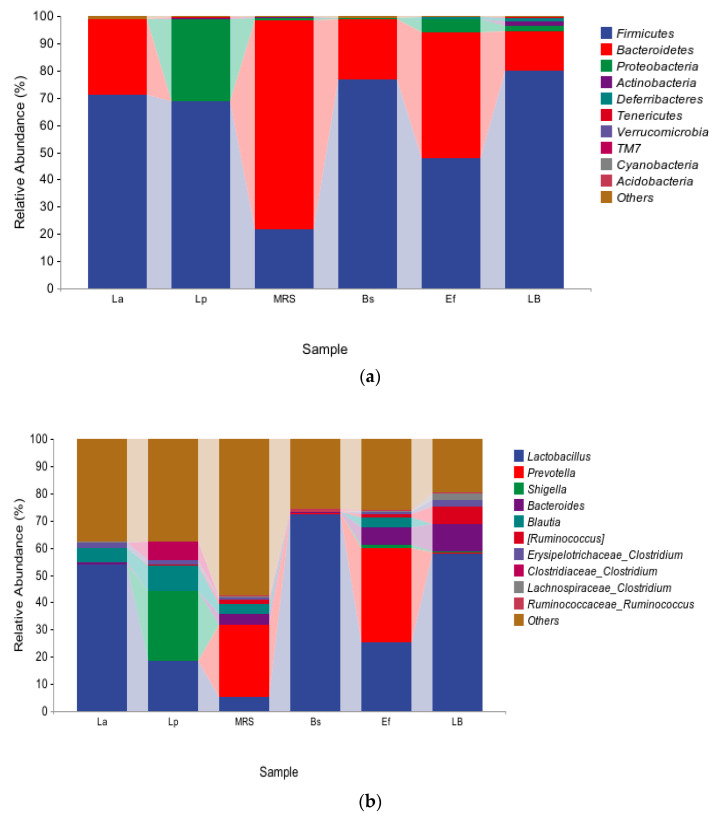
Distribution of gut microbiota: (**a**) species composition at the phylum level; (**b**) species composition at the genus level.

To study the effects caused by the four probiotics on the gut microbiota in mice, the top ten species with relative proportions at the phylum level and the genus level were selected, and a small number of species were classified as other species to evaluate the distribution of gut microbiota composition in mice. The species composition at the phylum level is shown in Figure 3a. Firmicutes and Bacteroidetes were dominant in the La group, and Firmicutes and Proteobacteria were dominant in the Lp group. In addition, the proportion of Proteobacteria was significantly increased in the Lp group compared with the control MRS group (*p* < 0.01). Firmicutes and Bacteroidetes were dominant in the Bs group; Firmicutes, Bacteroidetes, and Proteobacteria were dominant in the Ef group. Compared with the control LB group, Firmicutes, Bacteroidetes, and Proteobacteria in the Bs and Ef groups did not change significantly (*p* > 0.05). A few Bacteroidetes, Actinobacteria, and Deferribacteria were detected in the LB group, where Actinobacteria and Deferribacteres were significantly higher than those in the Bs and Ef groups (*p* < 0.01).

The species composition at the genus level is shown in Figure 3b. At the genus level, the distribution of the species in the six groups varied greatly. *Lactobacillus* was present in all six groups. *Lactobacillus*, *Bacteroides*, *Blautia*, *Lachnospiraceae Clostridium*, and *Erysipelotrichaceae Clostridium* were dominant in the La group. *Lactobacillus*, *Shigella*, *Blautia*, *Ruminococcus*, *Erysipelotrichaceae Clostridium*, and *Clostridiaceae Clostridium* were dominant in the Lp group. Compared with the control MRS group, *Lactobacillus* and *Lachnospiraceae Clostridium* in the La group were significantly higher (*p* < 0.01) and *Bacteroides* was significantly lower (*p* < 0.01). *Lactobacillus*, *Shigella*, Blautia, and *Clostridiaceae Clostridium* in the Lp group were significantly higher (*p* < 0.01), and *Ruminococcus* was significantly lower (*p* < 0.01). *Lactobacillus* and *Ruminococcaceae Ruminococcus* were dominant in the Bs group. *Lactobacillus*, *Prevotella*, *Shigella*, *Bacteroides*, *Blautia*, *Ruminococcus*, and *Erysipelotrichaceae Clostridium* were dominant in the Ef group. Compared with the control LB group, *Ruminococcaceae Ruminococcus* in the Bs group was significantly higher (*p* < 0.01), and *Prevotella*, *Bacteroides*, *Ruminococcus*, *Erysipelotrichaceae Clostridium*, and *Lachnospiraceae Clostridium* were significantly lower (*p* < 0.01). *Prevotella*, *Shigella*, *Blautia*, and *Ruminococcaceae Ruminococcus* in the Ef group were significantly higher (*p* < 0.01) and *Lactobacillus*, *Ruminococcus*, *Erysipelotrichaceae Clostridium*, and *Lachnospiraceae Clostridium* were significantly lower (*p* < 0.01).

Compared with the four experimental groups, *Lactobacillus* was significantly higher in the La and Bs groups than in the other two groups (*p* < 0.01). *Blautia* and *Erysipelotrichaceae Clostridium* in the La, Lp, and Ef groups were significantly higher than those in the Bs group (*p* < 0.01). *Bacteroides* and *Prevotella* had the highest proportion in the Ef group. *Blautia* had the highest proportion in the Lp group. *Shigella* in the Lp and Ef groups were significantly higher than those in the other two groups (*p* < 0.01), and *Shigella* was higher in the Lp group. *Clostridiaceae Clostridium* in the Lp group was significantly higher than in the other three groups (*p* < 0.01). *Lactobacillus* accounted for the highest proportion in the Bs group.

### 3.5. α-Diversity Analysis

**Figure 4:**
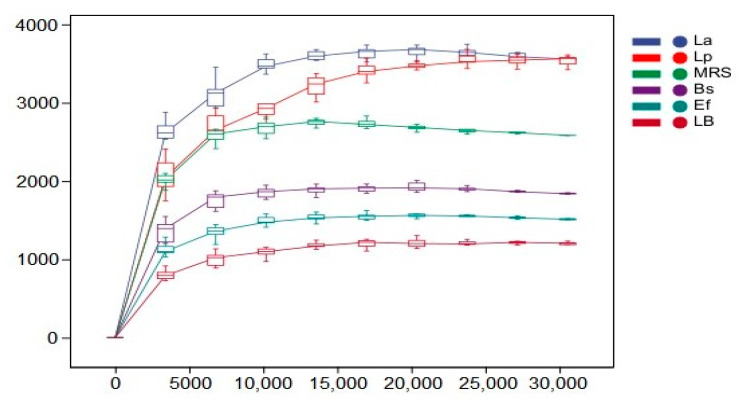
Dilution curves of the six sets of samples. Horizontal coordinates indicate the depth of sequencing, and vertical coordinates indicate the total number of species detected in the samples.

The rarefaction curves indicate the magnitude of the effect of sequencing depth on the diversity of the observed samples. The results are shown in Figure 4. With the increase in sequencing depth, the rarefaction curves of all six groups leveled off. The species diversity of all groups reached saturation, and no new ASV/OTU could be detected by continuing to increase the sequencing depth.

In order to assess the effect of four probiotics on the diversity and richness of the intestinal microbial community in mice, this study characterized the richness by the Chao1 index and the diversity by the Shannon and Simpson indices, and the individual indices of the samples are shown in Table 4. The mean Chao1 indices of the Bs, Ef, and LB groups were 1839.56, 1512.04, and 1214.77, respectively; the mean Shannon indices of the Lp, La, and MRS groups were 8.06856, 7.7823, and 8.39776, respectively; the mean Shannon indices of the Bs, Ef, and LB groups were 6.62015, 6.2814, and 6.237, respectively. The mean Simpson indices of the Lp, La, and MRS groups were 0.958022, 0.961972, and 0.964692, respectively, and the mean Simpson indices of the Bs, Ef, and LB groups were 0.88648, 0.904921, and 0.901051, respectively.

Relatively high Chao1, Shannon, and Simpson indices indicated high bacterial richness and diversity. In this study, the abundance of the La and Lp groups was significantly higher than in the control MRS group (*p* < 0.01). The diversity was not significantly different from the MRS group (*p* > 0.05). The abundance of Bs and Ef groups was significantly higher than the control LB group (*p* < 0.01). The diversity was not significantly different from the LB group (*p* > 0.05).

Compared with the four test groups, there was no significant difference between the La and Lp groups (*p* > 0.05). The richness of the Bs group was significantly higher than that of the Ef group (*p* < 0.01). Both the La group and the Lp group were significantly higher in richness and diversity than the Bs and Ef groups (*p* < 0.01).

### 3.6. β-Diversity Analysis

**Figure 5:**
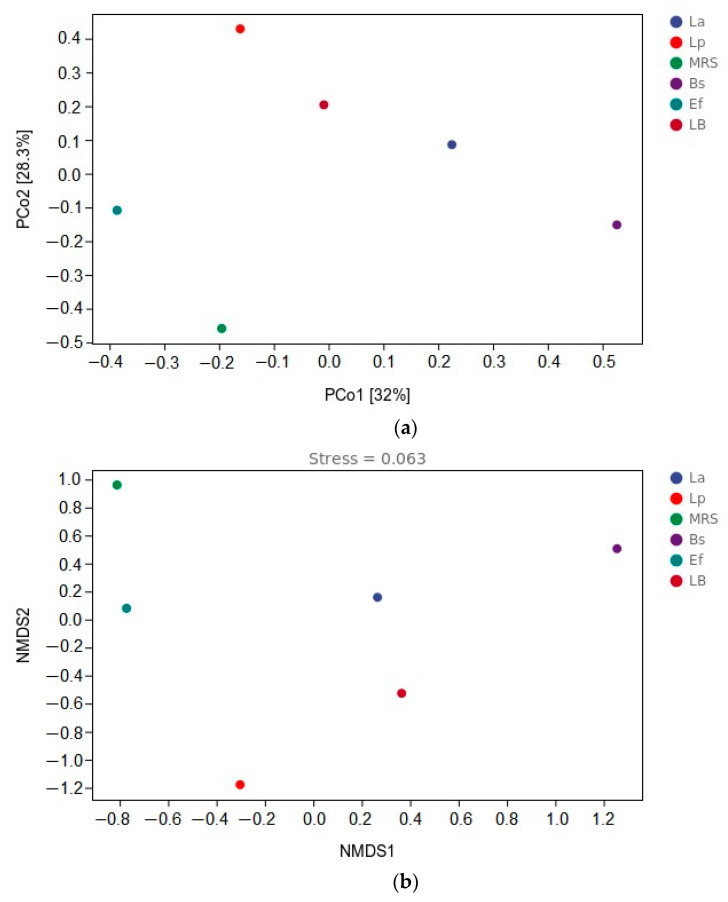
β-diversity analysis: (**a**) distance matrix and PCoA analysis; (**b**) β-diversity analysis and NMDS analysis.

To show the variability and similarity of microbial communities among the six groups of samples, this study used the principal co-ordinates analysis (PCoA) method and NMDS analysis. PCoA was used to expand the sample distance matrix in the low dimensional space after projection and to retain the distance relationship of the original samples to the maximum, as shown in Figure 5a. PCo1 and PCo2 accounted for 32% and 28.3% of the total variation, respectively. In the PCoA analysis, the coordinates of the six groups in the distance matrix were: La group (0.225, 0.086); Lp group (−0.161, 0.429); MRS group (−0.195, −0.458); Bs group (0.526, −0.151); Ef group (−0.386, (*p* < 0.05) −0.108); LB group (−0.008, −0.204). There were different clusters in all six groups of samples, and the distance between the six groups of samples was large. NMDS uses rank ordering, and the farther the distance between two points, the greater the difference between the microbial communities in the two samples, and vice versa, as shown in Figure 5b. In the NMDS analysis, the coordinates of the six groups in the distance matrix were: La group (0.265, 0.158); Lp group (−0.302, (*p* < 0.05). −1.178); MRS group (−0.811, 0.961); Bs group (1.255, 0.506); Ef group (−0.771, 0.080); LB group (0.365, −0.527). The greater distance between the six groups of samples in the NMDS analysis indicates that the difference in community composition is more significant (*p* < 0.01).

## 4. Discussion

According to the available studies, animal gut microbiota mainly includes bacteria, fungi, archaea, protozoa and viruses, which maintain the gut microecological balance in the host and interact with the host, thus influencing the host’s physiology and health [25]. The balance of gut microecology is closely related to the cardiovascular, neurological, immune, and metabolic systems of the host, and it is particularly important to maintain the composition and structure of the gut microbiota and to preserve its diversity [26]. Therefore, the aim of this study was to evaluate the effects of *L. acidophilus*, *L. plantarum*, *B. subtilis*, and *E. faecalis* on the structure and diversity of the gut microbiota in mice.

The results of this study show that. *L. acidophilus* can increase the proportion of Firmicutes, *Lactobacillus*, *Erysipelotrichaceae Clostridium*, and *Lachnospiraceae Clostridium* in the gut and reduce the proportion of *Bacteroides*. *L. plantarum* can increase the proportion of Firmicutes, Proteobacteria, *Lactobacillus*, *Shigella*, *Blautia*, and *Clostridiaceae Clostridium* in the gut and reduce the proportion of *Ruminococcus*. *B. subtilis* can increase the proportion of *Lactobacillus* and *Ruminococcaceae Ruminococcus*, and reduce the proportion of *Prevotella*, *Bacteroides*, *Ruminococcus*, *Erysipelotrichaceae Clostridium*, and *Lachnospiraceae Clostridium*. *E. faecalis* can increase the proportion of *Prevotella*, *Shigella*, *Blautia,* and *Ruminococcaceae Ruminococcus* and reduce the proportion of *Lactobacillus* and *Lachnospirace Clostridium*.

*L. acidophilus, L. plantarum, B. subtilis*, and *E. faecalis* could significantly increase the richness of the gut microbiota in mice. The effects of *L. acidophilus* and *L. plantarum* were more obvious. In addition, the four strains did not significantly affect the diversity of the gut microbiota in mice. The colony structure was greatly affected by the four species.

In previous studies, probiotic strains have been shown to be able to maintain the stability of the gut microbiota and interact with the intestinal microbiota by competing for nutrients, such as oxygen, through antagonism and cross-feeding in the gut [27]. The use of probiotics can reshape gut microbiota composition and improve microbial metabolism [28]. The four probiotics used in the present study altered the structure of the gut microbiota, which is consistent with the results of other studies. Several studies have shown that L. acidophilus increased the abundance of Firmicutes and *Lactobacillus* and decreased the abundance of Bacteroidetes, *Vibrio spp*., and *Ruminococcus* [29]; in other studies, *L. plantarum* increased the abundance of Firmicutes, Proteobacteria, and *Lactobacillus* and decreased the abundance of Bacteroidetes, [30,31]; *B. subtilis* can increase the abundance of Firmicutes, Bacteroidetes, and *Lactobacillus* [32]; *E. faecalis* can increase the abundance of Bacteroidetes and *Ruminococcus* and decrease the abundance of Proteobacteria [33]. This is similar to the results observed in the present study, where all four probiotics affected gut microbiota diversity differently, with significant changes in abundance (*p* < 0.01). In this study, although the four probiotics added did not have a significant effect on flora diversity, it was found that the four experimental groups differed significantly in their flora structure when analyzed between the groups.

In the present study, the increase in the abundance of Firmicutes may be related to the increase in beneficial bacterial species, such as Lactobacillus [34]. Notably, in the Bs group, the abundance of Firmicutes increased, which may be related to the consumption of oxygen by *B. subtilis* after colonization and the formation of an anaerobic environment, resulting in an increase in the abundance of anaerobionts such as *Lactobacillus*. In general, Firmicutes are associated with the ratio of Bacteroidetes and the susceptibility to disease states [35], but in the present study, the abundance of Bacteroidetes was reduced in the Lp group and Proteobacteria took the place of the original Bacteroidetes, making this ratio significant, while the mice did not exhibit significant disease, so, as a result, remained in a healthy state. Meanwhile, Actinobacteria and Deferribacteres appeared in the control LB group, so B. subtilis and *E. faecalis* may reduce the abundance of these two clades.

In addition, the present study found different variations at the genus level, with increased abundance of *Shigella* [36] in the Lp, Ef, and LB groups, and both in vivo [37,38] and in vitro [39,40] tests demonstrated that L. plantarum and *E. faecalis* could inhibit the growth of Shigella and alleviate the symptoms caused by *Shigella*, which differed significantly from the results of the present study, probably due to the use of *Blautia*, which is a newly discovered potential probiotic, the abundance of which is influenced by some prebiotics [41]. In this study, the increase in the abundance of *Blautia* may be influenced by *L. acidophilus*, *L. plantarum*, and *E. faecalis*; *B. subtilis* did not increase its abundance, and whether its growth is influenced by the growth of B. subtilis needs to be studied. *Prevotella* is abundant in the body as a key player in the balance between health and disease [42]. It has been shown that *L. acidophilus* can increase the abundance of *Prevotella* [29], which is contrary to the results of the present study; *L. plantarum* can decrease the abundance of *Prevotella* and *Bacteroides* [43,44], and *B. subtilis* and *E. faecalis* can increase the abundance of *Prevotella* [45,46]. All of these results are similar to the present study.

## 5. Conclusions

In this study, four probiotics: *L. acidophilus*, *L. plantarum*, *B. subtilis*, and *E. faecalis*, were gavaged into mice. The study showed that the four probiotics exerted different effects on the structure and richness of the gut microbiota in the intestines of the mice, and the study elucidated the mechanism of probiotic interactions in the intestine, which further provided a strong basis for the preparation of probiotics and theory regarding their targets.

## Figures and Tables

**Table 1:** Information on strains used in the experiment.

Number.	Strain	Source	Preservation Location
1	*L. plantarum*	Separated from mink manure in August 2017	Laboratory of Veterinary Microbiology and Immunology, College of Veterinary Medicine, Qingdao Agricultural University
2	*L. acidophilus*	Separated from mink manure in August 2017	Laboratory of Veterinary Microbiology and Immunology, College of Veterinary Medicine, Qingdao Agricultural University
3	*B. subtilis*	Separated from mink manure in August 2017	Laboratory of Veterinary Microbiology and Immunology, College of Veterinary Medicine, Qingdao Agricultural University
4	*E.* *faecalis*	Separated from mink manure in August 2017	Laboratory of Veterinary Microbiology and Immunology, College of Veterinary Medicine, Qingdao Agricultural University

**Table 2:** Bacterial concentration results.

	Time	6 h	8 h	10 h	12 h	14 h
Strains						
*L. plantarum*		6.01 × 10^8^	1.72 × 10^9^	2.34 × 10^9^	2.58 × 10^9^	2.62 × 10^9^
*L. acidophilus*		2.98 × 10^8^	1.35 × 10^9^	2.35 × 10^9^	3.55 × 10^9^	3.59 × 10^9^
*B. subtilis*		1.78 × 10^9^	2.28 × 10^9^	2.62 × 10^9^	2.65 × 10^9^	2.66 × 10^9^
*E.* *faecalis*		2.20 × 10^9^	2.61 × 10^9^	2.82 × 10^9^	2.90 × 10^9^	2.91 × 10^9^

Colony count formula:$\;\mathrm{Colony}\;\mathrm{concentration}=\mathrm{Bacterial}\;\mathrm{turbidity}\;\mathrm{value}\times 6.2\times 10^{9}$. Unit: CFU/mL.

**Table 3:** Sample sequencing statistics.

Sample ID	Input	Filtered	Denoised	Merged	Non-Chimeric	Non-Singleton
La	77,695	71,285	69,469	63,712	44,755	42,656
Lp	132,321	122,785	119,571	109,512	72,120	70,366
MRS	57,511	53,107	51,734	47,739	34,229	32,166
Bs	57,891	53,649	52,800	50,650	36,812	35,819
Ef	56,746	52,825	51,550	47,408	36,800	36,000
LB	77,804	73,483	72,382	69,136	49,334	48,793

In the table, “Sample ID” represents each group and sequentially represents the six groups of *L. acidophilus*, *L. plantarum*, MRS broth, *B. subtilis*, *E*. *faecalis*, and LB broth by gavage.

**Table 4:** α-diversity index.

Sample	Chao1	Shannon	Simpson
La	3555.3	8.06856	0.958022
Lp	3532.69	7.7823	0.961972
MRS	2582.61	8.39776	0.964692
Bs	1839.56	6.62015	0.88648
Ef	1512.04	6.2814	0.904921
LB	1214.77	6.23755	0.901051

In the table, “Sample” represents each group and sequentially represents the six groups of *L. acidophilus*, *L. plantarum*, MRS broth, *B. subtilis*, *E. faecalis*, and LB broth by gavage.

## Data Availability

The sequence data from this study have been uploaded to the NCBI database. BioProject: SUB12706298, BioSample: SUB12706389, and SRA: SUB12707064.
